# Anxiety symptoms and preventive measures during the COVID-19 outbreak in Taiwan

**DOI:** 10.1186/s12888-020-02786-8

**Published:** 2020-07-16

**Authors:** Li Ping Wong, Chia-Chun Hung, Haridah Alias, Tony Szu-Hsien Lee

**Affiliations:** 1 Centre for Epidemiology and Evidence-Based Practice, Department of Social and Preventive Medicine, Faculty of Medicine, University of Malaya 50603, Kuala Lumpur, Malaysia; 2 Institute of Brain Science, National Yang Ming University, Taipei, Taiwan; 3 Bali Psychiatric Center, Ministry of Health and Welfare, New Taipei City, Taiwan; 4 Department of Health Promotion and Health Education, National Taiwan Normal University, Taipei, Taiwan

**Keywords:** COVID-19, STAI-6, Preventive measures, Public anxiety, Taiwan

## Abstract

**Background:**

It is hypothesized that anxiety and behavioral responses are intense at the beginning of an epidemic. The objective of this study was to investigate anxiety symptoms and use of preventive measures against COVID-19. The study also compared the association between preventive measures and anxiety symptoms during the week immediately preceding the study and those symptoms and measures at the beginning of the outbreak.

**Methods:**

A cross-sectional population survey using an online questionnaire commenced on 14 February 2020. The study participants were residents of Taiwan ages 20 to 70 years. The 6-item state version of the State–Trait Anxiety Inventory (STAI-6) was used to assess anxiety symptoms. The questions about preventive measures asked participants about their personal protection, cough etiquette, contact precautions, voluntary quarantine, and prompt reporting. Multivariable logistic regression was used to determine the factors influencing an increase in the preventive measures scores.

**Results:**

Of a total of 3555 completed responses, a total of 52.1% (95% confidence interval [CI] 50.4–53.7) of the respondents reported moderate to severe levels of anxiety symptoms in the past week, whereas 48.8% (95%CI 47.2–50.5) reported moderate to severe anxiety symptoms at the beginning of the outbreak. With a higher score indicating greater anxiety, the median scores for anxiety symptoms in the past week and at the beginning of the outbreak were 46.7 (IQR [interquartile range] 36.7–53.3) and 43.3 (IQR 36.7–53.3), respectively. The median scores for the preventive measures taken in the past week and at the beginning of the outbreak were 26.0 (IQR 21.0–30.0) and 24.0 (IQR 19.0–28.0), respectively, out of a maximum score of 36. In the multivariable analysis, an increased anxiety symptom score from the beginning of the outbreak to the past week (adjusted OR = 7.38, 95%CI 6.28–8.66) was a strongly significant determinant of an increased preventive measures score in the past week compared with the score at the beginning of the outbreak.

**Conclusions:**

Anxiety and preventive measures scores were high and increased with the epidemic rate. Higher anxiety was associated with an increased use of preventive measures against COVID-19.

## Background

On 9 January 2020, a novel coronavirus was detected as the causative agent linked to cases of pneumonia reported in Wuhan, China and the genome sequence was made public [1]. Subsequently, the rapid spread of the outbreak across Wuhan and, at a later date, outside mainland China prompted the Chinese government to take immediate action to lockdown the epicenter and several nearby cities in the province of Hubei. The rapid spread of the novel coronavirus caused much anxiety and stigma and on 11 February 2020, the World Health Organization (WHO) declared the official name of the new coronavirus disease to be COVID-19 [2].

The severity and highly contagious nature of COVID-19 have sparked concern worldwide and especially in the countries neighbouring China. The close proximity of Taiwan to China, coupled with the high frequency of cross-border travel, has placed Taiwan as one of the most highly threatened countries, among the other neighbouring countries in Asia, in relation to a COVID-19 outbreak. Taiwan’s government learned from the severe acute respiratory syndrome (SARS) epidemic in 2003, as such, the country has been on constant alert for another epidemic. The Taiwanese government has raised the levels of alert and worry about a potential COVID-19 outbreak in the country. The country has also issued an advisory warning against travel to China. At a later date, various measures were undertaken by the Taiwan government to contain the spread of the COVID-19 virus, including travel bans, and quarantine for those who had recently been to China and who had close contact with confirmed cases of COVID-19. Despite these measures, the first case of COVID-19 in Taiwan was reported on 21 January 2020. On 8 February, the number of confirmed cases in Taiwan had increased to 18. On 16 February, the first coronavirus death was reported in Taiwan, and the total number of confirmed cases was 20. The first death, from a local transmission in Taiwan, also marked the fifth fatality outside mainland China [3]. Taiwan has been listed by the Centers for Disease Control (CDC) and Prevention as one of the countries with an apparent community spread [4].

The COVID-19 outbreak threatens not only the life of those infected but also the psychological health of the affected community [5]. The lessons learned from previous outbreaks such as Ebola [6], SARS [7], and the 2009 influenza A (H1N1) [8, 9] have led to a worldwide recognition of the importance of addressing morbidities related to mental health associated with the outbreak. The impact of such morbidities during a disease outbreak is crucial, not only because it results in a significant weakening in social and other important areas of functioning, including failure in preventive measures [10, 11], but also because excessive mental distress may have a number of biological effects, including reduced cell-mediated immunity and increased inflammatory processes [12, 13]. Recently, a review of global evidence reported that quarantine during previous infectious disease outbreaks such as SARS, Ebola, H1N1 influenza, and Middle East respiratory syndrome resulted in serious psychological consequences with potentially long-lasting effects [14]. Thus, it is important for the general population, in particular people who are under home quarantine or social isolation, to reduce the distress associated with the outbreak in order to maintain an optimal state of psychosocial well-being.

Considering the severe psychological effect of the COVID-19 outbreak, it is tremendously important to assess the lay public’s psychobehavioural response to the outbreak in order to develop and implement effective preventive and emotional regulation control strategies. An important element of outbreak management is an appropriate psychobehavioural response to preventive measures so as to minimize the spread of infection. Experiences from the previous SARS epidemic highlights the importance of appropriate behavioural responses in controlling an epidemic [15, 16].

Because of the importance of determining the level of psychological distress and understanding the psychobehavioural consequences of COVID-19 in the general public while the epidemic is still ongoing, our main objective in this study was to investigate the anxiety symptoms caused by COVID-19, and use of preventive measures against COVID-19, among the general public. We also aimed to compare the anxiety symptoms and preventive measures during the week immediately prior to the study, while the outbreak was still on the rise, and at the time the COVID-19 outbreak was first reported in China. We hypothesized that the preventive practices and level of anxiety in the public increased as the number of COVID-19 cases increased. Further anxiety has also been hypothesized to act as a trigger to preventive practices during disease outbreak [10], thus, we also investigated the influence of anxiety on the use of preventive practices.

## Methods

### Study design and participants

We commenced a cross-sectional, web-based anonymous survey using an online questionnaire on 14 February 2020. In Taiwan, nearly 73% of the population is between the ages of 15 and 65 years [17]. The legal age of adulthood in the Civil Code of Taiwan is 20 years. Therefore, we surveyed Taiwan residents 20 years old and above. Participants were informed that their participation was voluntary, and consent was implied by the completion of the questionnaire. For this online survey, consent obtained from the participants was verbal and the Institutional Review Board has waived written (signed) consent. Our survey ended on 16 February 2020, immediately after the report of the first death from the COVID-19 virus in Taiwan.

### Procedures

The snowballing sampling technique was used to recruit the participants. The researchers used social networks, namely Facebook and LINE (the most popular messaging app in Taiwan) to circulate the survey link to all members on their contact lists. When participants anonymously completed the survey, they were encouraged to disseminate the survey link to all their contacts with a thank you note at the end. The questionnaire was developed in English and then translated into Mandarin. Local experts validated the content of the questionnaire, after which it was pilot tested. The survey consisted of three sections, which assessed (1) demographic background, (2) anxiety symptoms, and (3) use of preventive measures. The survey took approximately 10 min to complete.

#### Anxiety symptoms

The 6-item state version of the State–Trait Anxiety Inventory (STAI-6), which assesses anxiety symptoms, was adapted from previous studies for use in this study [18, 19]. The STAI-6 has been shown to be highly correlated with the 20-item STAI, and all internal consistency reliabilities are greater than 0.90 [20]. The STAI-6 has been used both as a self-administered and online questionnaires [21–23]. The Chinese version of the STAI was used in this survey [24]. The participants rated the frequency of experiencing six emotional states, namely being calm, tense, upset, relaxed, content, and worried, in relation to the current COVID-19 outbreak. A 4-point scale was used (1 = not at all, 2 = somewhat, 3 = moderately, 4 = very much). The scores on the three positively-worded items were reverse coded. The participants were asked to rate each of the anxiety symptoms during the *past week* (PW)-starting on February 8 and during the *beginning of the outbreak* (BO)- January 23 (the day of the Wuhan lockdown) to February 8*.* The anxiety symptoms during the PW are defined as the anxiety level between 8 February and the time of the survey. The anxiety level during the BO is defined as the anxiety symptoms experienced from the date of the Wuhan lockdown (23 January 2020) until 8 February 2020. The total summed scores for the PW and BO periods were prorated (multiplied by 20/6) in order to obtain scores that were comparable with those from the full 20-item STAI (range from 20 to 80) [18]. Scores of 44 or above were defined as indicating moderate to severe symptoms [10, 25, 26].

#### Preventive measures

Questions assessing use of preventive measures were developed specifically for this study (Additional file 1). Preventive measures were assessed using questions that asked the participants about their personal protection (four items), cough etiquette (four items), contact precautions (two items), voluntary quarantine (one item), and prompt reporting (one item). The response options were on a 4-point Likert scale, with the items scored as 0 (rarely), 1 (occasionally), 2 (often), and 3 (always). The participants were asked to answer each question about the preventive measures they had carried out during the PW and during the BO*.* As with the anxiety levels, the preventive measures during the PW are defined as preventive measures carried out between 8 February and the time of the survey, and preventive measures during the BO are the measures carried out from the date of the Wuhan lockdown (23 January 2020) until 8 February 2020. The total scores for the preventive measures for each period were obtained by summing across all the questions. The possible score ranges from 0 to 36, with higher scores indicating higher use of preventive measures.

### Ethical considerations

The study protocol was approved by the Research Ethics Committee of the National Taiwan Normal University (no. 202002HS007). Informed consent was obtained using an online consent form that the participant had to actively agree to and all participants were 20 years old and above. This method of consent was approved by the ethics committee.

### Statistical analyses

The reliability of the anxiety symptoms and preventive measure scores were evaluated by assessing their internal consistency. The 12 items for preventive measures had a reliability (Cronbach’s α) of 0.891. Multivariable logistic regression was used to determine the factors influencing an increase in the preventive measures score (1 = increase in use of preventive measures score over time; 0 = no increase or a decrease in use of preventive measures over time). Variables that were significant by a chi-square (χ^2^) test were selected for multivariable logistic regression analysis and included in the model, using a simultaneous forced-entry method. Odds ratios (OR), 95% confidence intervals (95%CI), and *p* values were calculated for each independent variable. The model fit was assessed using the Hosmer–Lemeshow goodness-of-fit test [27]. All statistical analyses were performed using the Statistical Package for the Social Sciences, version 20.0 (IBM Corp., Armonk, NY, USA). A *p* < value of less than 0.05 was considered statistically significant.

## Results

Of a total of 3555 complete responses, the majority were from the North district of Taiwan (66.3%), females (78.2%), and ages 31–40 years (36.9%) or 41–50 years (28.4%). The great majority had either a college degree (58.2%) or postgraduate degree (34.8%). A summary of the characteristics of the respondents is provided in the first and second columns of Table 1.

**Table 1:** Participant characteristics and anxiety symptom score past week and beginning of the outbreak (*N* = 3555)

			Past week (score ≥ 44) (***n*** = 1852)		Beginning of the outbreak (score ≥ 44) (***n*** = 1738)		Differences (Past week-beginning of the outbreak
Socio demographic characteristics	N	%(95% CI)	n	%(95% CI)	n	% (95% CI)	% (95% CI)
Age group (years)							
20–30	468	13.2 (12.1–14.3)	228	48.7 (44.2–53.2)	241	51.5 (47.0–56.0)	−2.8 (1.6–4.7)
31–40	1313	36.9 (35.3–38.5)	750	57.1 (54.4–59.8)	705	53.7 (51.0–56.4)	3.4 (2.6–4.6)
41–50	1008	28.4 (26.9–29.9)	521	51.7 (48.6–54.8)	469	46.5 (43.5–49.6)	5.2 (4.0–6.7)
50–70	766	21.5 (20.2–22.9)	353	46.1 (42.6–49.6)	323	42.2 (38.7–45.7)	3.9 (2.8–5.5)
Gender							
Male	775	21.8 (20.5–23.2)	317	40.9 (37.5–44.4)	296	38.2 (34.8–41.7)	2.7 (1.8–4.1)
Female	2780	78.2 (76.8–79.5)	1535	55.2 (53.4–57.1)	1442	51.9 (50.0–53.7)	3.3 (2.7–4.1)
Highest educational level							
High school and below	250	7.0 (6.2–7.9)	140	56.0 (49.6–62.2)	125	50.0 (43.9–56.2)	6.0 (3.7–9.7)
College	2069	58.2 (56.6–59.8)	1136	54.9 (52.8–57.0)	1060	51.2 (49.1–53.4)	3.7 (2.9–4.6)
Graduate and above	1236	34.8 (33.2–36.4)	576	46.6 (43.8–49.4)	553	44.7 (42.0–47.5)	1.9 (1.2–2.8)
Average monthly household income (NT$)							
< 20,000	528	14.9 (13.7–16.1)	287	54.4 (50.1–58.6)	257	48.7 (44.4–52.9)	5.7 (4.0–8.0)
20,000-50,000	1440	40.5 (38.9–42.1)	783	54.4 (51.8–56.9)	743	51.6 (49.0–54.2)	2.8 (2.1–3.8)
50,000-100,000	1251	35.2 (33.6–36.8)	647	51.7 (49.0–54.5)	608	48.6 (45.8–51.4)	3.1 (2.3–4.2)
> 100,000	336	9.5 (8.5–10.5)	135	40.2 (35.1–45.5)	130	38.7 (33.6–44.0)	1.5 (0.6–3.4)
Location							
North district	2358	66.3 (64.7–67.9)	1252	53.1 (51.1–55.1)	1164	49.4 (47.3–51.4)	3.7 (3.0–4.6)
Central district	599	16.8 (15.6–18.1)	317	52.9 (48.9–56.9)	298	49.7 (45.8–53.7)	3.2 (2.0–4.9)
South district	484	13.6 (12.5–14.8)	239	49.4 (45.0–53.8)	233	48.1 (43.7–52.6)	1.3 (0.6–2.7)

### Anxiety symptoms

A total of 52.1% (95%CI 50.4–53.7) of the respondents reported moderate to severe anxiety symptoms on the STAI for the PW period, whereas 48.8% (95%CI 47.2–50.5) reported moderate to severe anxiety symptoms for the BO period (Table 1). The highest proportions of moderate to severe cases of anxiety during the PW period were respondents ages 31–40 years (57.1%; 95%CI 54.4–59.8), female (55.2%; 95%CI 53.4–57.1), having a high school education or below (56.0%; 95%CI 49.6–62.2), and from the North district (53.1%; 95%CI 51.1–55.1). Similar proportions of respondents with average household incomes below NT$20,000 (54.4%; 95%CI 50.1–58.6) and between NT$20,000 and NT$50,000 (54.4%; 95%CI 51.8–56.9) reported moderate to severe cases of anxiety for the PW period.

The highest proportion of moderate to severe cases of anxiety during the BO period were respondents ages 31–40 years (53.7%; 95%CI 51.0–56.4), female (51.9%; 95%CI 50.0–53.7), having a college education (51.2%; 95%CI 49.1–53.4), and with a household income between NT$20,000 and NT$50,000 (51.6%; 95%CI 49.0–54.2). Similar proportions of respondents from the North (49.4%; 95%CI 47.3–51.4) and Central (49.7%; 95%CI 45.8–53.7) districts reported moderate to severe anxiety during the BO period. On the whole, respondents who were older, male, whose highest educational level was a college degree and above, in the higher income group and from the South or East districts reported the lower levels of anxiety symptoms during both the PW and the BO periods.

The greatest increase in the proportion of moderate to severe cases of anxiety from the PW to the BO periods was from those in the 41–50 age group (5.2%; 95%CI 4.0–6.7), and this value was statistically significant. In contrast, there was a decrease in the proportion with moderate to severe anxiety (− 2.8%; 95%CI − 9.2–3.6) among the respondents in the 20–30 age group. Female participants showed a significantly higher increase in the proportion of moderate to severe cases of anxiety (3.3%; 95%CI 2.7–4.1) than male participants (2.7%; 95%CI 1.8–4.1). The highest increase in the proportion with moderate to severe anxiety came from respondents whose highest educational level was high school or below (6.0%; 95%CI 3.7–9.7) and whose income was below NT$20,000 (5.7%; 95%CI 4.0–8.0). Increases in the proportions of moderate to severe anxiety levels were greater in respondents from the North (3.7%; 95%CI 3.0–4.6) and Central (3.2%; 95%CI 2.0–4.9) districts than from other districts.

The difference in mean STAI score for the PW period (46.2, SD = 11.68) and the BO period (45.0, SD = 12.13) was statistically significant (Wilcoxon signed rank test = 6.414, *p* < 0.001). The median scores for anxiety symptoms during the PW and the BO periods were 46.7 (IQR 36.7–53.3) and 43.3 (IQR 36.7–53.3), respectively. The scale for both scores ranges from 20 to 80. In total, 1229 (34.6%; 95%CI 33.0–36.2) had increases in their anxiety scores from the PW to the BO periods (PW score > BO score), and 2326 (65.4%; 95%CI 63.9–67.0) recorded a similar or lower anxiety score (PW score ≤ BO score).

### Preventive measures

A summary of responses in relation to use of preventive measures during the PW and BO periods is shown in Table 2. During the PW period, the highest proportion of use of personal protective measures was for respondents who frequently washed their hands with soap (49.0%; 95%CI 47.3–50.7), followed by those who wore a mask (31.8%; 95%CI 30.3–33.4). As for cough etiquette, the most commonly practised preventive measures were disposing of tissues immediately (80.1%; 95%CI 78.9–81.4) and covering one’s mouth and nose when coughing or sneezing (65.2%; 95%CI 63.6–66.7). Always performing hand hygiene was reported by a high proportion of the participants (60.3%; 95%CI 58.6–61.9). Over a third of respondents reported that they always avoided proximity with others (34.4%; 95%CI 32.8–36.0) and avoided gathering in groups (41.2%; 95%CI 39.5–42.8). Nearly two-thirds reported that they always voluntarily quarantined themselves if they were feeling unwell (61.6%; 95%CI 60.0–63.2), but fewer than half said they would always report promptly to health authorities if they were feeling unwell (45.4%; 95%CI 43.8–47.1).

**Table 2:** Differences in preventive measures scores between past week and beginning of outbreak (*N* = 3555)

Preventive measures	Always practice - past week		Always practice - beginning of outbreak		Differences (past week-beginning of outbreak)
	n	% (95 CI%)	n	% (95 CI%)	% (95 CI%)
***Personal protection***					
Wear a mask	1132	31.8 (30.3–33.4)	802	22.6 (21.2–24.0)	9.2 (8.4–10.3)
Eye protection	447	12.6 (11.5–13.7)	381	10.7 (9.7–11.8)	1.9 (1.5–2.4)
Wash hand frequently with soap	1742	49.0 (47.3–50.7)	1359	38.2 (36.6–39.8)	10.8 (9.8–11.8)
Avoid touching your eyes, nose, and mouth	1059	29.8 (28.3–31.3)	777	21.9 (20.5–23.3)	7.9 (7.1–8.9)
***Respiratory etiquette/cough Etiquette***					
Cover mouth and nose with tissue when cough or sneeze.	2317	65.2 (63.6–66.7)	2212	62.2 (60.6–63.8)	3.0 (2.4–3.6)
Dispose tissue paper immediately after coughing or sneezing	2848	80.1 (78.9–81.4)	2363	66.5 (64.9–68.0)	13.6 (12.6–14.8)
Wash hand immediately after coughing or sneezing.	1115	31.4 (29.8–32.9)	968	27.2 (25.8–28.7)	4.2 (3.5–4.9)
Perform hand hygiene (e.g., hand washing with or antiseptic handwash) after having contact with respiratory secretions or contaminated objects	2142	60.3 (58.6–61.9)	1904	53.6 (51.9–55.2)	6.7 (5.9–7.6)
***Contact precautious***					
Avoid proximity (closeness) with other people	1222	34.4 (32.8–36.0)	961	27.0 (25.6–28.5)	7.3 (6.5–8.2)
Avoid group gathering	1463	41.2 (39.5–42.8)	1096	30.8 (29.3–32.4)	10.3 (9.4–11.4)
***Voluntary quarantine***					
If I am feeling unwell, I distance myself from others	2191	61.6 (60.0–63.2)	2016	56.7 (55.1–58.3)	4.9 (4.3–5.7)
***Prompt reporting***					
If I am feeling unwell, I will immediately declare my symptoms to the authority/healthcare providers	1615	45.4 (43.8–47.1)	1410	39.7 (38.0–41.3)	5.8 (5.1–6.6)

Similar findings were reported for preventive measures during the BO period, with the highest proportion reporting that they always disposed of tissues immediately after coughing and sneezing (66.5%; 95%CI 64.9–68.0), followed by those who reported covering their mouths and noses when coughing or sneezing (62.2%; 95%CI 60.6–63.8). The proportion who practised contact precautions was relatively low. A comparison of the proportions who always practised particular preventive measures during the PW and the BO periods shows that disposing of tissues immediately after coughing and sneezing recorded the highest increase (13.6%; 95%CI 12.6–14.8), followed by avoiding group gatherings (10.3%; 95%CI 9.4–11.4) and frequently washing hands (10.8%; 95%CI 9.8–11.8). The increases were lower for using eye protection (1.9%; 95%CI 1.5–2.4) and covering one’s mouth and nose when coughing or sneezing (3.0%; 95%CI 2.4–3.6).

The difference in the mean total scores for preventive measures during the PW (25.4, SD = 5.85) and the BO periods (23.4, SD = 6.56) was statistically significant (Wilcoxon signed rank test = 23.917, *p* < 0.001). The median scores for preventive measures during the PW and the BO periods were 26.0 (IQR 21.0–30.0) and 24.0 (IQR 19.0–28.0), respectively. The observed total scores for use of preventive measures ranged from 2 to 36 for the PW period and 0–36 for the BO period. A total of 1696 (47.7, 95%CI: 46.1–49.4) respondents had an increase in their total preventive measures scores from the PW to the BO periods, and 1859 (52.3, 95%CI: 50.6–53.9) had similar or lower total preventive measures scores.

Table 3 shows results of the univariate and multivariable analyses of the factors associated with a higher total preventive measures scores in the PW periods than in the BO period. Having a higher anxiety score for the PW than for the BO period (adjusted OR = 7.38, 95%CI 6.28–8.66) was a strong significant determinant of an increase in the preventive measure score from the BO to the PW periods. Respondents with a college-level education (adjusted OR = 1.58, 95% 1.17–2.13) and graduate level education or above (adjusted OR = 1.74, 95% 1.28–2.38) were more likely to have an increased preventive measure scores from the BO to the PW periods than those whose highest education level was high school or below.

**Table 3:** Factors associated with differences in total preventive measure scores (*N* = 3555)

	Univariable analysis				Multivariable analysis^**a**^
		Differences in total preventive measures scores			Differences in total preventive measures scores Past week > score beginning outbreak vs Past week ≤ score beginning of outbreak
Covariates	Frequency (%)	Past week > score beginning of outbreak (***n*** = 1696)	Past week ≤ score beginning of outbreak (***n*** = 1859)	***P***	OR (95% CI)
**Socio demographic characteristics**					
Age group (years)					
20–30	468 (13.2)	227 (48.5)	241 (51.5)		
31–40	1313 (36.9)	638 (48.6)	675 (51.4)	0.735	
41–50	1008 (28.4)	477 (47.3)	531 (52.7)		
50–70	766 (21.5)	354 (46.2)	412 (53.8)		
Gender					
Male	775 (21.8)	372 (48.0)	403 (52.0)	0.871	
Female	2780 (78.2)	1324 (47.6)	1456 (52.4)		
Highest educational level					
High school and below	250 (7.0)	96 (38.4)	154 (61.6)		Reference
College	2069 (58.2)	994 (48.0)	1075 (52.0)	0.008	1.58 (1.17–2.13)**
Graduate and above	1236 (34.8)	606 (49.0)	630 (51.0)		1.74 (1.28–2.38)***
Average monthly household income (NT$)					
< 20,000	528 (14.9)	232 (43.9)	296 (56.1)		
20,000-50,000	1440 (40.5)	688 (47.8)	752 (52.2)	0.138	
50,000-100,000	1251 (35.2)	622 (49.7)	629 (50.3)		
> 100,000	336 (9.5)	154 (45.8)	182 (54.2)		
Location					
North district	2358 (66.3)	1128 (47.8)	1230 (52.2)		
Central district	599 (16.8)	277 (46.2)	322 (53.8)	0.838	
South district	484 (13.6)	234 (48.3)	250 (51.7)		
East district	114 (3.2)	57 (50.0)	57 (50.0)		
**Anxiety symptom score**					
Differences in score between past 1 week and beginning the outbreak					
Score past 1 week ≤ score beginning the outbreak	2326 (65.4)	744 (32.0)	1582 (68.0)	*p* < 0.001	Reference
Score past 1 week > score beginning the outbreak	1229 (34.6)	952 (77.5)	277 (22.5)		7.38 (6.28–8.66)***

**p < 0.01, ***p < 0.001

^a^Hosmer–Lemeshow test, chi-square: 4.812, *p*-value: 0.307; Nagelkerke *R*^*2*^: 0.240

## Discussion

Approximately 3 weeks after the widespread news of the lockdown of the epicenter of COVID-19, we found the alarming result that over half (52.1%) of lay members of the Taiwanese public who participated in the survey had moderate to severe anxiety. The anxiety level of our study sample is similar to that reported in mainland China. A recently published study conducted in mainland China at the same time as our study reported that 53.8% of the people in China rated the psychological impact of the outbreak as moderate or severe [28]. Those in the younger age groups, females, and those with less education and a lower income were found to report more anxiety symptoms. This provides valuable information to allow public health authorities and stakeholders to coordinate their targeted mental health interventions. Similarly, other recently published studies conducted in mainland China during the COVID-19 pandemic also found higher anxiety among females and younger people [28, 29]. Higher anxiety among females has been reported in SARS outbreaks [16]. This greater psychological impact of disease outbreaks on females corresponds to many psychiatric epidemiology findings, namely, that women being significantly more likely than men to develop an anxiety disorder when exposed to traumatic events [30, 31]. Of important note, the reason why the younger respondents in our study expressed a higher degree of anxiety is unknown, and this warrants further investigation. Future studies should focus on the consequences of the infodemic of false information about COVID-19 circulating on social media and of the social anxiety in Taiwan. The huge amount of information about the novel coronavirus circulating on social media has been reported to spark anxiety, fear and panic in users [32]. It is also noteworthy that in our study the older age group reported the lowest anxiety levels during the time of the interview and the beginning of the outbreak; however, they reported a greater increase in anxiety over time. This could perhaps be due to an increase of reporting in the news media nationwide of evidence of elderly people’s elevated risk of serious illness and death from the new coronavirus.

Despite it being widely circulated in the news media that older adults affected by COVID*-*19 likely have a higher risk of complications and mortality, and the recent large epidemiological data from the Chinese Centre for Disease Control and Prevention that shows that those above 60 years of age accounted for 44.1% of confirmed cases in Wuhan [33], our study’s participants in the older age group appear to have a lower level of anxiety. Further studies are warranted to determine whether the low anxiety stems from a lack of knowledge or the respondents being out-of-touch with current media interventions. The exponential increase in confirmed COVID-19 cases in mainland China following the lockdown, as well as the increasing numbers of cases outside China, including in Taiwan, has sparked global concern and likewise an increase in anxiety among the participants in this study. Further, the advisory warning against travel, including border control from the air and sea, and daily press briefings by the ministry of health and welfare of the Central Epidemic Command Center (CECC) of Taiwan [34] perhaps also caused or exacerbated stress in the community. Concurrent with the relatively low anxiety levels among those with higher education and higher incomes, the increase in anxiety was also lowest among the group with the higher socio-economic status. The underlying reasons for these socio-economic disparities in anxiety also warrant further investigation.

Since the early phase of the COVID-19 pandemic, people in Taiwan have been constantly provided health information that encourages the practice of proper handwashing, social distancing, and cough etiquette through mass media and billboards in public areas. This perhaps resulted in good preventive measures being adopted by the study participants, as evidenced by the median scores of 25 and 23, out of a possible score of 36, for the past week and the beginning of the outbreak, respectively. Further, nearly half of them reported improved preventive practices in the past week compared to the beginning of the outbreak. We also found a considerable increase in use of the preventive measures in hand washing, cough etiquette, and contact precaution, which perhaps explains why, as of February 16, only 20 confirmed COVID-19 cases have been reported in Taiwan, despite it being in close geographical proximity to mainland China and having a high volume of travellers to and from mainland China prior to the outbreak. Nonetheless, the findings highlight the need to improve contact precautions, since the proportion of respondents who were avoiding proximity and group gatherings was low at both the beginning of the outbreak and in the past week. Multivariable analyses revealed that participants with higher education levels reported a greater increase in the use of preventive measures. A greater increase in anxiety symptoms was associated with a greater increase in preventive practices. This finding is in congruence with previous findings from the SARS outbreak in Hong Kong where a positive dose-response gradient between anxiety level and adoption of personal protective measures was found [15], suggesting that the right level of anxiety, not so high as to result in a detrimental health impact, can perhaps help to stimulate practices that prevent an epidemic.

The current study has several limitations. The first limitation is that the responses were based on self-reports and may be subject to self-report and recall biases. Second, the online survey with snowballing sampling resulted in a sample that was lack of representativeness of the general population. In Taiwan, females account for 50.4% of the total population, and 46.7% have completed tertiary education. High school graduates and below account for 35.5 and 17.8%, respectively. The Northern region of Taiwan accounts for nearly half (45.6%) of the total Taiwan population [35]. It is notable that the study had an over-representation of female and highly educated participants, and an unequal distribution of respondents among the districts of Taiwan. The current total Taiwanese population is estimated at 23,816,775. Taipei, the capital city, and its surrounding metropolitan area are on the Northern part of the island; this resulted in a large proportion of our study participants coming from the Northern region. Nevertheless, during the crisis period of the outbreak, the snowballing method, which uses LINE and Facebook, the largest social media platform in Taiwan, was extremely effective in achieving a wide coverage of participants. Despite the limitations, the study contributes tremendously to the understanding of the psychological well-being and psychobehavioural responses of the general public in Taiwan associated with COVID-19 while the epidemic is still on-going.

## Conclusion

Anxiety and the use preventive measures increased along with the increase in the COVID-19 epidemic rate. Higher anxiety was associated with greater use of preventive measures against COVID-19. It is of the utmost urgency that public health interventions be carried out to reach the identified groups of people with poor adherence to preventive measures. Considering the extremely contagious nature of COVID*-*19, a slight lack of compliance by even a small portion of the population may have grave consequences and contribute to the continued spread of the coronavirus. The demographics disparities in psychological effects and psychobehavioural responses to the outbreak found in this study provide important information for developing and implementing targeted preventive and emotional regulation control strategies.

## Supplementary information

**Additional file 1.** Questions assessing preventive measures against COVID-19.

## Data Availability

The datasets used and analyzed during the current study are available from the corresponding author on reasonable request.

## Abbreviations

BO: Beginning of outbreak
CI: Confidence Interval
COVID-19: Coronavirus disease-2019
H1N1: Influenza A
NT$: New Taiwan Dollar
PW: Past week
SARS: Severe acute respiratory syndrome
STAI-6: State–Trait Anxiety Inventory
WHO: World Health Organization
