# Supplementary material for: Anxiety symptoms and preventive measures during the COVID-19 outbreak in Taiwan
Source: BMC Psychiatry. 2020 Jul 16;20:376. doi: 10.1186/s12888-020-02786-8 (PMC7364127; doi:10.1186/s12888-020-02786-8)
Supplement: Supplementary file 1 — Additional file 1. Questions assessing preventive measures against COVID-19. [file 12888_2020_2786_MOESM1_ESM.docx]

Questions assessing prevention measures against COVID-19

| **Prevention measures** | **Option answer** |
| --- | --- |
|  |  |
| ***Personal protection*** |  |
| Wear a mask | [ ] Rarely [ ] Occasionally [ ] Often [ ] Always |
| Eye protection | [ ] Rarely [ ] Occasionally [ ] Often [ ] Always |
| Wash hand frequently with soap | [ ] Rarely [ ] Occasionally [ ] Often [ ] Always |
| Avoid touching your eyes, nose, and mouth | [ ] Rarely [ ] Occasionally [ ] Often [ ] Always |
| ***Etiquette/Cough Etiquette*** |  |
| Cover mouth and nose with tissue when cough or sneeze. | [ ] Rarely [ ] Occasionally [ ] Often [ ] Always |
| Dispose tissue paper immediately after coughing or sneezing | [ ] Rarely [ ] Occasionally [ ] Often [ ] Always |
| Wash hand immediately after coughing or sneezing. | [ ] Rarely [ ] Occasionally [ ] Often [ ] Always |
| Perform hand hygiene (e.g., hand washing with or antiseptic handwash) after having contact with respiratory secretions or contaminated objects | [ ] Rarely [ ] Occasionally [ ] Often [ ] Always |
| ***Contact precautious*** |  |
| Avoid proximity (closeness) with other people | [ ] Rarely [ ] Occasionally [ ] Often [ ] Always |
| Avoid group gathering | [ ] Rarely [ ] Occasionally [ ] Often [ ] Always |
| ***Voluntary quarantine*** | [ ] Rarely [ ] Occasionally [ ] Often [ ] Always |
| If I am feeling unwell, I distance myself from others | [ ] Rarely [ ] Occasionally [ ] Often [ ] Always |
| ***Prompt reporting*** |  |
| If I am feeling unwell, I will immediately declare my symptoms to the authority/healthcare providers | [ ] Rarely [ ] Occasionally [ ] Often [ ] Always |
